# Packet Loss Characterization Using Cross Layer Information and HMM for Wi-Fi Networks

**DOI:** 10.3390/s22228592

**Published:** 2022-11-08

**Authors:** Carlos Alexandre Gouvea da Silva, Carlos Marcelo Pedroso

**Affiliations:** Department of Electrical Engineering, Federal University of Paraná, Curitiba 80060-000, Paraná, Brazil

**Keywords:** Hidden Markov Model, IEEE 802.11, packet loss models, SNR, wireless communication

## Abstract

Packet loss is a major problem for wireless networks and has significant effects on the perceived quality of many internet services. Packet loss models are used to understand the behavior of packet losses caused by several reasons, e.g., interferences, coexistence, fading, collisions, and insufficient/excessive memory buffers. Among these, the Gilbert-Elliot (GE) model, based on a two-state Markov chain, is the most used model in communication networks. However, research has proven that the GE model is inadequate to represent the real behavior of packet losses in Wi-Fi networks. In this last category, variables of a single network layer are used, usually the physical one. In this article, we propose a new packet loss model for Wi-Fi that simultaneously considers the temporal behavior of losses and the variables that describe the state of the network. In addition, the model uses two important variables, the signal-to-noise ratio and the network occupation, which none of the packet loss models available for Wi-Fi networks simultaneously take into account. The proposed model uses the well-known Hidden Markov Model (HMM), which facilitates training and forecasting. At each state of HMM, the burst-length of losses is characterized using probability distributions. The model was evaluated by comparing computer simulation and real data samples for validation, and using the log-log complementary distribution of burst-length. We compared the proposed model with competing models through the analysis of mean square error (MSE) using a validation sample collected from a real network. Results demonstrated that the proposed model outperforms the currently available models for packet loss in Wi-Fi networks.

## 1. Introduction

Current reports estimate that the number of devices connected to mobile and wireless networks are increasing considerably. The number of Wi-Fi hotspots will increase from 169 million in 2018 to 628 million in 2023 [1]. The Wi-Fi connection speeds of mobile devices should triple by 2023, when the average connection speed of Wi-Fi networks (30.3 Mbps in 2018) will exceed 91.6 Mbps [1]. This increase is expected due to the development of new technologies, devices, and improvements in the communication systems currently in operation.

Wireless local area networks (WLAN) are computer networks that link devices using wireless communication within a limited area, such as in homes, industries, hotels, and restaurants, among others. IEEE 802.11, known as Wireless Fidelity (Wi-Fi), is part of the IEEE 802 set of LAN protocols. It specifies the set of media access control (MAC) and physical layer (PHY) [2], and is currently the standard for WLAN [3].

Packet loss occurs when a packet cannot correctly reach its destination node. The loss can have several reasons and may be classified into three types: (1) physical layer losses, due to problems in the transmission channel; (2) MAC layer losses, due to competition for channel access; or (3) network congestion losses, due to insufficient link rate, equipments with small buffers, or bufferbloat (a problem caused by large buffers) [4]. Then, the performance and evaluation of WLAN depends on different quality factors at different layers of the network protocol stack, such as at the PHY and MAC layers of communication [3].

The currently available packet loss models for Wi-Fi networks can be classified into two categories: (1) use of time series observation of a sequence of packet discards in a real network, through a mathematical relationship applied to past data, and (2) use of relationships between the packet loss and variables that describe the state of the network, such as the signal-to-noise ratio and the transmission rate, among others [4]. A packet loss model is an abstraction or a simplified representation of the loss behavior of a real system. Gilbert-Elliot (GE) [5,6] is the most used model, even though it is already quite old. It is based on a two-state Markov chain [7], called Good (G) and Bad (B), in which the probability of a loss is respectively given by 1−k and 1−h. The probability of transition between states G and B is given by *p* and *q*, respectively [4]. However, the GE model fails to represent packet loss in IEEE 802.11 networks due to the existence of heavy-tailed run [8] and also because, in real scenarios, the losses occur in bursts [9,10,11]. In wireless communication networks, packet loss models consider singly PHY layer parameters such as SNR, or MAC layer parameters [4]. In other cases, the losses are only characterized by analytical models based in a temporal series unrelated to the observations of the state of the network. Hidden Markov Models (HMM) have been used as an alternative for modeling and analyzing different behaviors in wireless networks [12,13,14], including techniques of channel selection [15]. An HMM is a stochastic model formed by two structures, in which the first is an unknown stochastic process impossible to observe directly and may only be inferred by a second observable process [16,17].

In this article, we propose a new packet loss model based on HMM for IEEE 802.11b/g/n networks considering the signal-to-noise ratio (SNR) and the channel occupation as a sequence of observable data. For HMM training and validation, samples were collected from the Wi-Fi network of the Engineering Department at the Federal University of Paraná, Brazil. To identify the number of states, we used data clustering techniques. For each of the states, the burst loss length (BLL) was fitted to probability distributions. The performance evaluation was carried out through the comparison between computer simulations and real traffic samples. The main competing models were also simulated. The results demonstrate that the proposed model outperforms the currently available models.

The main contributions of this work can be summarized as follows: (I) We made and presented many tests with different variables existing in WiFi networks that are classified as irrelevant or redundant to model the behavior of packet losses, thus allowing a reduction in the number of variables simplifying the parameterization and use of the model. These tests reduce the requirement of only two variables for an accuracy packet loss model. (II) A new packet loss model for IEEE 802.11b/g/n networks based on HMM is proposed where the size of burst losses is classified into four states, and each state is modeled using heavy-tailed distributions. (III) The proposed model considers two variables, SNR and channel occupation. Current models do not consider these two variables simultaneously to model losses. (IV) It is also demonstrated that for WiFi networks under ideal transmission conditions in the physical layer, where the SNR is high, the losses have a great influence on the channel occupation. In this case, considering only the SNR for loss models is flawed because other important factors are not considered, that is, the occupation of the channel in the link layer.

The remainder of this paper is organized as follows: Section 2 presents the main packet loss models for Wi-Fi networks; Section 3 presents the process of collecting, identifying, and defining observable data that were used in HMM training; the proposed model and the performance evaluation are presented in Section 4. Finally, Section 5 presents the conclusions.

## 2. Related Works

The Gilbert-Elliot model is one of the most popular in packet loss modeling [18]. However, two-state Markov models fail to represent and fit long-term statistics of packet loss [4,8]. In order to improve the accuracy of models, the HMM has been gaining great prominence to model the behavior and characteristics of wireless networks and also addresses several aspects and analyses of the network [13].

Cardoso and Rezende have proposed the use of HMM to model the packet loss in Wi-Fi networks using three states with two structures: general (HMM3g, with transitions between every pair of states) and birth-death (HMM3bd transitions only between adjacent states) [11]. Computer simulations demonstrate that HMM3bd overperforms the Gilbert-Elliot model in terms of autocorrelation function (ACF) and complementary cumulative distribution function (CCDF) of traffic bursts, whereas HMM3g only presets small improvements. However, even with some improvements, HMM3bd is not yet sufficient to adequately describe the loss process, and it is necessary to increase the number of states to improve accuracy. According to the authors, the model’s only drawback is that the optimal number of states can vary from trace to trace [11].

Another approach using HMM to model packet loss in Wi-Fi networks was conducted by Salih et al. [19]. The proposed model is a double embedded process (DEPHMM) that uses the number of losses as a criterion for establishing the number of states in the model. The packet loss ratio varies according to the BLL, which consequently makes the parametrization of the DEPHMM more complex. The performance evaluation considers traces extracted only from simulation runs. DEPHMM is compared with the Deterministic Process Based Generative Model (DPBGM) [20,21] and the Finite State Markov Chain (FSMC) [22]. Results indicate that DEPHMM is capable of constructing binary packet error sequences with burst error statistics that closely match the reference traces.

Hartwell and Fapojuwo [23] propose the use of a five-state HMM to model packet loss in Wi-Fi networks. In this model, the state transition matrix defines the probability of the channel proceeding to each different state for every received frame interval. This way, from the observation of the received packets in a time interval, Viterbi’s algorithm will indicate the most probable state. The model uses a set of sample data for parametrization and performance evaluation. Models with 2, 3, 4, and 5 states were tested. Results demonstrate that high-order models trained with the Baum-Welch algorithm outperform the Gilbert-Elliot model.

Russ and Haghani [8] present a packet loss model for IEEE 802.11g based on a combination of the classic Gilbert-Elliot and a long-tail model. The authors suggest that the BLL can be expressed by two different models, considering the consecutive number of packets lost as *n* (i.e., for n≤3 it is better to use the Gilbert-Elliot model while for n>3 the use of a long heavy-tailed distribution has better results). The authors also suggest a deep investigation to determine a heavy-tailed distribution applicable to longer bursts.

In this article, we propose the use of HMM in a different way from the ones that previously used it in the literature. We will determine which observable variables are relevant in the loss of packets and, through these variables, determine the most likely state the system is in. In addition, we will conduct a deeper study on the ideal number of states to be used.

Table 1 presents a comparison of related works available in the literature and the proposed new packet loss model. Moreover, this table demonstrates the contribution of proposed model that uses simultaneously two variables with HMM training. The other related works uses only one parameter, and any one case suggests using SNR or occupation network in order to train a HMM system.

## 3. Packet Loss Modeling with Cross Layer Information

**Figure 1:**
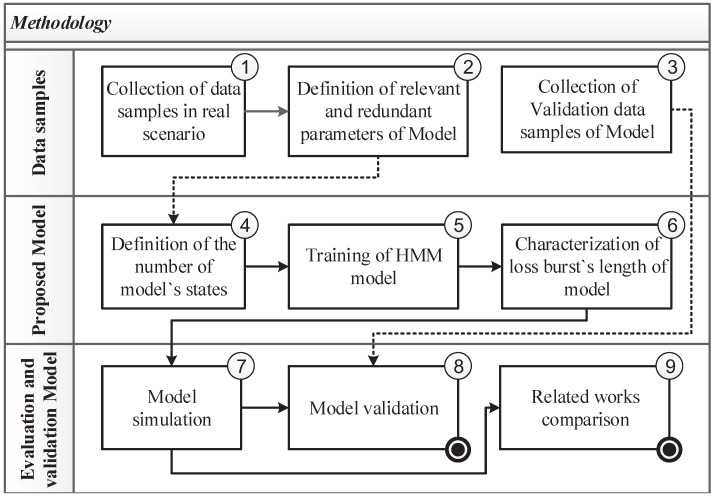
Methodology of this article.

The methodology used to develop the new packet loss model can be divided into three parts, as shown in Figure 1: (1) data sample collection and definition of relevant/redundant variables, (2) definition of the number of states, HMM training, and characterization of BLL, and (3) performance evaluation of the proposed model and comparison with related works. Each digital number 1 to 9 is explicated in the rest of the paper.

### 3.1. Measurement Setup and Data Collection

The measurement setup consisted of an indoor 802.11 network in the Department of Electrical Engineering at the Federal University of Paraná. The building quarters research laboratories, classrooms, teachers’ offices, and attendance offices. The Wi-Fi users consist of around 1300 people, including graduate and undergraduate students, teachers, research, technicians, and visitors. The Wi-Fi network uses 15 IEEE 802.11n access points spread throughout the building to provide wireless coverage, with overlapping channels in most of its locations.

The measurement setup consisted of an Access Point (AP) connected to a laptop that repeatedly sent out constant size, constant rate packets using the UDP protocol, acting as the source IP address. A computer connected to the AP using an uncongested Ethernet network was the destination IP address and responsible for recording the packets’ arrival time. Aside from the time of sent and received packets, additional information was collected, i.e., signal strength (dBm), transmission rate (Mbps), and channel quality. We estimated the delay and jitter of each packet received and used a Fluke AirCheck™Wi-Fi Tester to record other variables of interest, such as SNR, channel occupation, number of users in the channel, among others. The AP configuration setup is shown in Table 2.

Several samples were collected in different days and hours in order to capture a range of situations. Moreover, in order to increase data diversity, the laptop was moved to different points in the building during capture. The network was in constant use and presented a varied number of users connected to the AP or other adjacent APs that share the channel. According to Abraham et al. [28], it is very difficult to avoid the partial overlapping of Wi-Fi channels due to the limited number of orthogonal channels in IEEE 802.11 standards.

The sample consisted of 24,600 min (or 410 h) of traffic in different situations of network occupation, SNR, number of active users, and distance from the AP.

### 3.2. Variables of Interest

A model can use numerous variables to correlate and predict the behavior of a system [29]. In our case, there are at least 10 candidate variables to be used, but this number is too large to be considered in a statistical model. We used statistical correlation techniques to determine which variables are relevant, irrelevant, and which variables are dependent on each other.

The correlation model was used to identify the relevant parameters, using correlation tests based on Pearson coefficient. Pearson’s method uses a correlation coefficient (ρ) that can take values ranging from −1 to +1, where ρ=+1 indicates a perfect positive correlation between the two variables, ρ = −1 represents a perfect negative correlation, and ρ=0 indicates that the two variables do not depend on each other. The ρ coefficient is given by

(1) $$\rho =\frac{\sum_{i=1}^{n}\left(x_{i}-\bar{x}\right)\left(y_{i}-\bar{y}\right)}{\sqrt{\sum_{i=1}^{n}{\left(x_{i}-\bar{x}\right)}^{2}}\cdot \sqrt{\sum_{i=1}^{n}{\left(y_{i}-\bar{y}\right)}^{2}}}$$

where $x_{i}$ and $y_{i}$ are values of two paired variables, $\bar{x}$ e $\bar{y}$ are the variables’ average, and *n* is the sample size.

Variables with high correlation to each other are considered redundant, thus the number of input parameters can be reduced. Where variables present low correlation with the observed packet loss, they are considered irrelevant and can also be discarded.

Packet delay is the amount of time that a packet takes to reach the receiving end point, and jitter is the variation in the delay of received packets. In IEEE 802.11, the transmission rate relies on many factors such as channel bandwidth, number of spatial streams, guard interval, encoding rate, and type of modulation scheme. For SNR lower than 25 dB, we verified that delay and jitter were strongly correlated to SNR, with the correlation coefficient given by ρ=−0.9442 and ρ=−0.9026, respectively. For the same interval, the SNR and packet loss rate (PLR) presented a correlation of ρ=−0.6436. For SNR higher than 25 dB, packet losses showed a correlation of ρ=0.8222 and ρ=0.7953 in relation to the observed delay and jitter, respectively. Thus, there is a strong influence of the SNR in both the delay and the jitter on Wi-Fi networks. Since published papers have also demonstrate that both delay and jitter parameters are related to SNR [30,31,32,33], delay and jitter were considered redundant due to correlation with SNR.

The correlation between channel occupation and the number of users in the AP were tested in different intervals of the sample. We observed that a high occupancy channel is not related to the number of users connected, because connected users are not necessarily transmitting at any given time. Furthermore, a channel can have high occupancy with only one active user. When analyzing the correlation between channel occupation, the number of users, and the packet loss rate, it was possible to observe that the higher loss rates were predominant in high occupation levels. Correlation between the number of users and the loss rate was given by ρ=−0.0779 (not correlated); the channel occupation and the loss rate presented ρ=0.8162 (correlated). Thus, channel occupation was defined as a parameter for the model, while the number of users was not, since it was considered irrelevant.

## 4. Proposed Model

In a Markov chain, each state corresponds to an observable event [16]. The HMM is a class of probabilistic graphical model with state (hidden) variables, which are estimated through a sequence of output (observable) events or variables [34]. In hidden Markov models, the current state of the system is not directly observable. These models have wide application in speech recognition, DNA sequence, and video streaming client behavior, among others.

**Figure 2:**
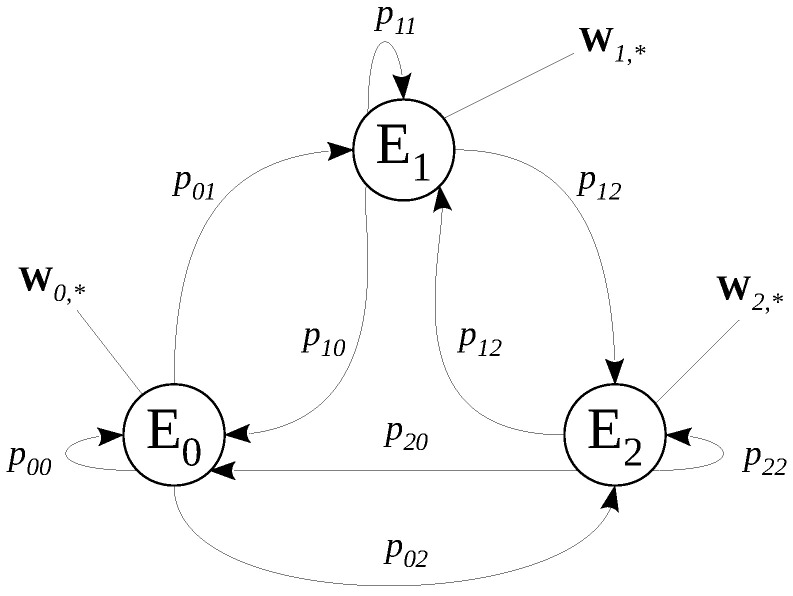
A three-state Hidden Markov Model.

Figure 2 exemplifies a three-state HMM, where $E_{k}$, with $k=\left\{0,1,2\right\}$, represents the hidden states; $p_{kj}$, with $j=\left\{0,1,2\right\}$ being the value for the transition probabilities from state $E_{k}$ to $E_{j}$, and vector $w_{k,*}=\left[w_{k0},w_{k1},\ldots ,w_{k(M-1)}\right]$ represents the probabilities of observation, also called emission probabilities, of the observable events m∈{0,1,…,M−1} in a given state $E_{k}$. The number of hidden states is given by *K* (also called HMM order). In our model, the observable event was defined by the tuple {SNR, channel occupation}.

Unsupervised HMM training was performed using the Baum-Welch algorithm. This process requires a sequence of observable events and, during training, the algorithm adjusts the state transition probabilities $p_{kj}$ and the emission probabilities $w_{k,*}$. The meaning of the states can only be carried out after training, analyzing the characteristics of the formed groups.

After training, the transition and emission probabilities are used to determine the most likely state using only observable events as input. In this work, the Viterbi algorithm was used to evaluate the most likely state. If the network training has been carried out in a channel occupation and SNR representative way, the parameters obtained can be applied in other network scenarios without the need for new training.

Another advantage of our approach is that it makes the system less dependent on the number of states because the observable events are configured by combinations between the SNR and the channel occupation. This contrasts with the related works, which use only the loss sequence as an observable event. When using the number of losses, the system tends to perform better as the number of states increases, which can be observed in practically all models that followed this approach.

### 4.1. Order Selection

According to Pohle et al. [35], conceptually, the order selection appears to be a simple model selection task, but in practice it remains a notoriously difficult challenge. Although the number of states can be empirically estimated, this approach reduces the accuracy of the model. Instead of the empirical approach, we used clustering techniques to select the HMM order. Clustering algorithms are usually applied to solve problems related to data mining [36]. Clustering is a technique used for grouping data, categorized into unsupervised and semi-supervised [37], with the main goal of classifying a set of data objects into several groups named clusters. The objects of a cluster must have high similarity to each other and must be dissimilar to the objects of other clusters [38]. Most well-known similarity measures, such as Euclidean, Minkowski, Hamming, and Jaccard distances, are only concerned with a single data point. However, we are interested in finding a distance measure between the collection of SNR and channel occupation points all correlated with each other in a very interesting way. Each cluster was created based on the best similarity of these nearby distances. There are, unsurprisingly, a myriad of methods for doing this, each applicable to a certain subset of problems, but it will be promoted to one in order to be using with HMM.

**Figure 3:**
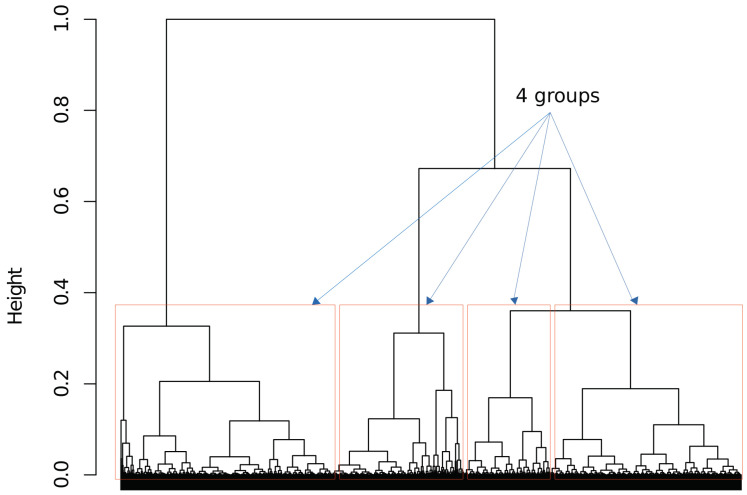
Dendrogram of SNR and channel occupation.

In this article, we used the Euclidean distance method, and the results are presented in a dendrogram to illustrate the arrangement of clusters, visually representing the hierarchical relationship between objects [39]. Euclidean distance generally seeks to compare time series directly, so that time series with similar shapes are assigned lower distances. The Euclidean distance is $d\left(A,B\right)=\sqrt{{\left(x_{2}-x_{1}\right)}^{2}+{\left(y_{2}-y_{1}\right)}^{2}}$, where points A and B are $A(x_{1},y_{1})$ and $B(x_{2},y_{2})$, respectively. Figure 3 presents the resulting dendrogram using the SNR and the channel occupation as objects. The level of similarity is measured along the vertical axis, in which long vertical lines (height) indicate higher similarity among each cluster’s data. Visually, it is possible to verify the existence of 3, 4, or 5 groups. HMM training also acts as a data classifier, and the quality of the classification can be assessed after training. The training of HMM with four states presented better results when compared with three or five states. Therefore, four states were used in the proposed model.

**Figure 4:**
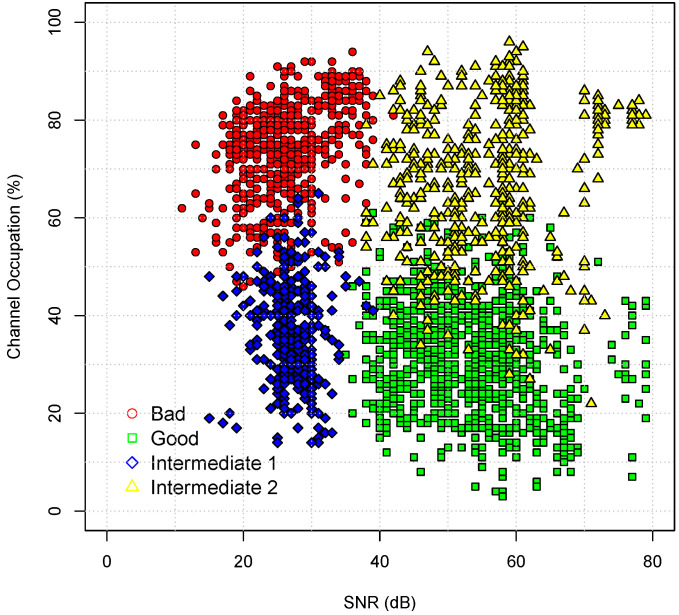
Identification states based on the HMM4.

Figure 4 illustrates the classification performed by the HMM training algorithm. Table 3 shows the classification of each state and their average PLR with the respective standard deviations. We classified the states as follows: good (G), bad (B), intermediate 1 (I1), and intermediate 2 (I2).

State B presents the highest average PLR (60.97%). Figure 4 shows that this state occurs in situations of high channel occupation and low SNR. State G is the best state, since it has the lowest average PLR (0.55%), the highest levels of SNR and lowest values of channel occupation. States I1 and I2 are considered intermediaries. State I1 was classified with low SNR and low channel occupation, with an average PLR of 2.02%. State I2 presents an average PLR of 12.78%, with high SNR and low channel occupation—the losses in this state are driven by the channel occupation, a characteristic which is not captured by the competing models.

HMM4 training process resulted in the probability transition matrix given by

$$\begin{array}{cc} & \begin{array}{cccc} B & G & I1 & I2 \end{array}\\ P=\begin{array}{c} B\\ G\\ I1\\ I2 \end{array} & \left(\begin{array}{cccc} 0.955 & 0.014 & 0.017 & 0.014\\ 0.002 & 0.959 & 0.013 & 0.027\\ 0.051 & 0.017 & 0.932 & 0.000\\ 0.014 & 0.061 & 0.000 & 0.925 \end{array}\right) \end{array}$$

The transition probability matrix indicates that the most likely event is to remain in the state. From state B, it is possible to reach all states with a similar probability. State G transitions with the probabilities of 0.2% to state B, 1.3% to state I1, and 2.7% to state I2. The probability of transition from state I1 to B is 5.1%, and 1.7% to G. It is important to note that state I1 does not reach state I2 directly, and state I2, which has the second highest PLR, does not directly reach state I1. The probability of transition from state I2 to state B is 1.4% and 6.1% to state G.

The matrix shows that the probability of staying in the same state is greater than 95% for either bad or good states, although there is the possibility of transitioning to all other states. As for states I1 and I2, the probability of remaining in the same state is greater than 92%, however, the possibility of transition between these two states is the lowest, as they only transition to bad and good states. The dynamics of state transition allows us to capture the temporal changes related to changes in the SNR and channel occupation, which will translate into a better adherence of the model to the empirical data.

The BLL, which is given by the number of losses until the next packet is received, was recorded for each state. Heavy-tailed distributions are used to model the BLL in wireless networks [8,25]. A random variable *X* has a heavy-tailed distribution if

(2) $$P\left(X>x\right)∼c.x^{-\alpha }$$

as x←∞, where α is the shape parameter and *c* is a positive constant.

The empirical probability distribution of BLL in each state was fitted and compared with several probability distributions, i.e., Pareto type II, Weibull, Log-Normal, Cauchy, and Log-Cauchy. The parameterization of each probability distribution was performed using estimators available in several libraries available in the R software. Among the parameter estimators used in this process, one can mention the Maximum Likelihood (MLE), Moment Matching (MLE), Quantile Matching (QME ) and Maximizing Goodness-of-fit Estimation (MGE). When verifying the cumulative distribution function (CDF) of the length of the loss bursts from the distributions used in the adherence verification process, it was observed that the Pareto Type II distribution presented better adherence in relation to the others that do not fitted very well. The use of the Pareto Type II distribution is already suggested in other works in the modeling of packet loss bursts [40,41,42].

A QQ-Plot is a scatterplot created by plotting two sets of quantiles against one other. If both sets come from the same distribution, a 45 degree line will be formed. Thus, we used the QQ-Plot to graphically compare the empirical BLL and theoretical distributions for all four hidden states.

**Figure 5:**
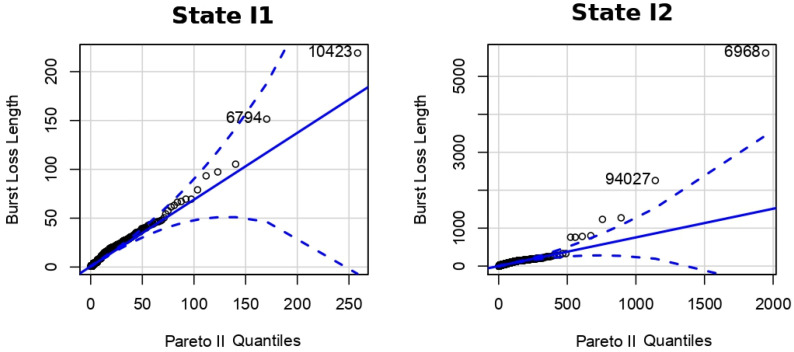
QQ-Plot for states I1 and I2.

The Pareto type II probability density function (PDF), in turn, is a heavy-tailed distribution given by

(3) $$Pr\left(X=x\right)=\frac{\lambda \alpha }{{\left(1-\lambda x\right)}^{\alpha +1}},\;\alpha >0,\lambda >0,x>0$$

where λ is the scale parameter and α is the shape parameter. Figure 5 shows the QQ-Plot comparing the BLL of states I1 and I2 with the Pareto type II probability distribution.

The continuous line in Figure 5 represents the perfect goodness of fit between the two variables. The dotted lines represent the limits for 95% confidence and the quantiles are plotted as circles. The observed packet BLL fitted very well to the Pareto type II.

However, due to the presence of spikes in the BLL of states G and B, the Pareto type II distribution was unable to fully characterize the empirical data. In both states, the Pareto type II distribution fitted correctly for BLL up to 40 and 400, respectively. In order to solve this problem, we used a combination of an exponential distribution and the Pareto type II distribution. The PDF of exponential distribution is given by:

(4) $$Pr\left(X=x\right)=\frac{1}{\mu }e^{-x/\mu },\;x>0,$$

where μ is the expected value.

**Figure 6:**
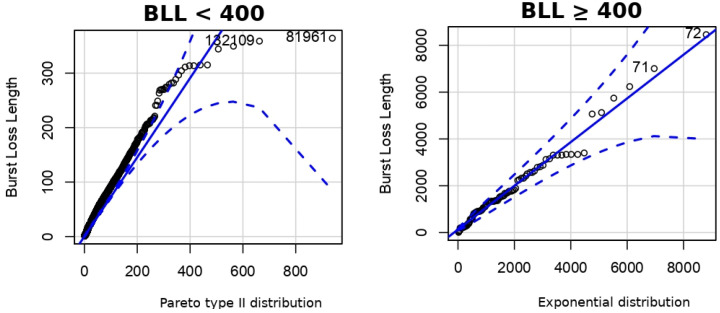
QQ-Plot for state G.

**Figure 7:**
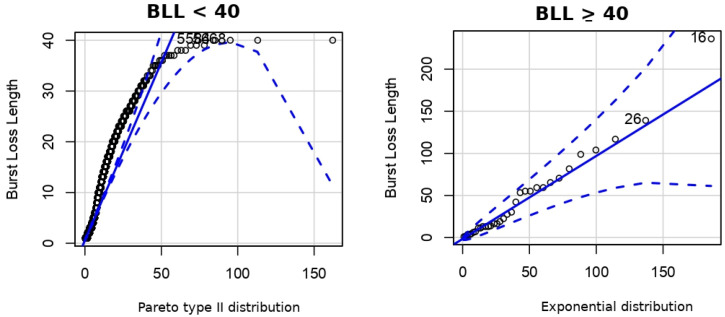
QQ-Plot for state B.

The Pareto type II distribution models the body of the distribution while the exponential distribution models the tail of states G and B. It is important to note that less than 1% of situations need to be modeled with the exponential distribution. Figure 6 and Figure 7 present the QQ-Plot for states G and B, respectively. The BLL fitted well using the aforementioned thresholds with the Pareto type II distribution and the exponential distribution for each state.

Table 4 presents the estimated values of Pareto type II and exponential distribution parameters for all states.

### 4.2. Performance Evaluation

The proposed model was evaluated using a validation data set not previously used in HMM training or goodness of fit tests. Each sample of validation data set has SNR, channel occupation, and the sequence of lost or received packets. The most probable state was estimated using the Viterbi algorithm [43], available in the statistical software R (R version 3.6.1) [44]. The proposed model was then used to simulate the packet loss using the previously obtained state transition probability matrix and the BLL probability distributions in each state. As the losses of the validation set are known, it is possible to compare the model with the empirical data.

**Figure 8:**
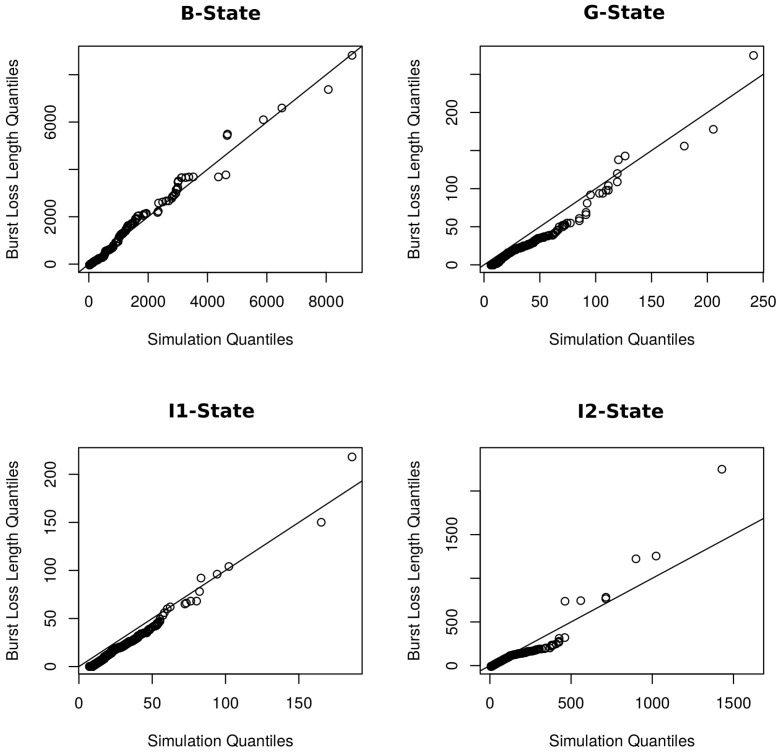
QQ-Plot of burst length of HMM 4 states and simulation results.

The results of this comparison are presented in Table 5, where the average BLL of the simulation and validation data set are similar. Additionally, we plotted the QQ-plot of empirical and simulated BLL, as shown in Figure 8. This figure shows that simulations data can accurately represent the empirical BLL. Simulated traces of states B, G, I1, and I2 fitted very well to the BLL when compared to empirical data.

**Figure 9:**
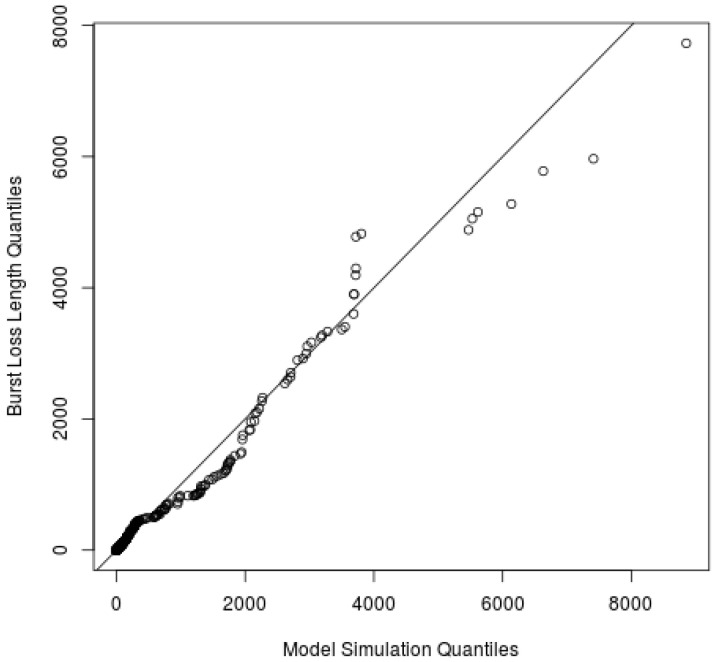
QQ-Plot of BLL of validation data set and simulation.

Figure 9 presents QQ-Plot of the validation data set and the computer simulation of the proposed model, considering all burst loss sequences. The result indicates that the model represents the real system’s behavior very well.

### 4.3. Competing Models Comparison

**Figure 10:**
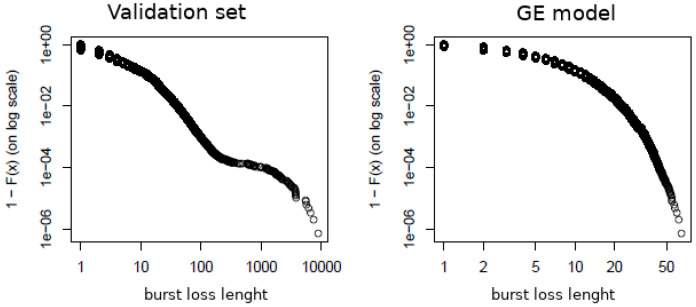
LLCD of BLL for the validation set and the GE model.

First, we compared the BLL observed in the validation set with the BLL generated by the GE model. The parametrization of the GE model was conducted using the same data from the validation set. In the GE model, the probability of transition between the bad and good states are p=0.0393 and q=0.1862, respectively for p=P(B|G) and q=P(G|B). Figure 10 plots the log–log complementary distribution (LLCD, 1−P(X≤x) in a log scale) for the BLL of the validation data set and the one generated by the GE model. The results demonstrate that the GE model cannot capture the behavior of the real system.

As proposed by Arauz and Krishnamurthy [24], we separated the samples using four SNR thresholds from 10 dB to 75 dB. Each level represents a state of the model and is modeled as a two-state Markov chain. The first state (10–26 dB) is the worst state, and the fourth state (60–75 dB) is the best one.

In the model proposed by Carvalho et al. [10], the BLL is modeled through a geometric series distribution. The parameter θ=0.936848 was estimated using the Maximum Likelihood Estimation (MLE). The GE model with substates proposed by Feng et al. [26] suggests a good state with a set of four adjacent states. The probability of the transition to a bad state is $p_{1}=0.009749,\;p_{2}=0.004928,\;p_{3}=0.002926,\;\mathrm{and}\;p_{4}=0.001855$.

An adaptation between the GE model and heavy-tailed distributions was proposed by Russ and Haghani [8]. The burst loss with a length lower or equal to three losses was simulated with the GE model (p=0.6240 and q=0.6216), while the burst loss greater than three losses was simulated with the Pareto type II distribution (shape=2.5192 and scale=19.7564).

The Finite State Markov Chain (FSMC) model presented by [27] was simulated with 4, 5, 7, 10, and 20 states, where the transition probability was estimated using the validation data set.

It was not possible to simulate the DEPHMM [19] and HMM3g [11] due to the lack of information about the configuration of observable events in the respective articles. We suspect that, in these cases, the only observable event is the series of packet loss, but the articles do not report this explicitly.

Table 6 presents a comparison of average BLL, maximum burst length, BLL standard deviation, Mean Square Error (MSE) of burst loss, and the validation set for all competing models described prior to the proposed model. Our model presents average BLL of 5.52, maximum burst length of 7728, and standard deviation of 29.75. All those values are close to the validation data set, thus, the proposed model significantly outperforms the existing ones. The second best model was the Russ and Haghani [8], which suggests the use of a heavy-tailed distribution to model burst lengths greater than three. Increasing the number of states of the FSMC model increases the maximum BLL; however, it also increases the standard deviation, which indicates that increasing the number of states is not enough to improve the performance of this model.

**Figure 11:**
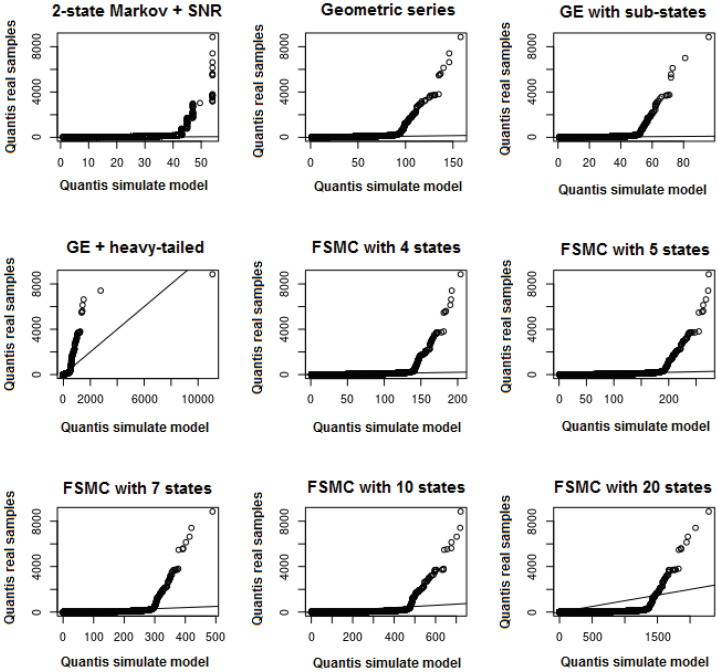
QQ-Plot: simulation model of related works with the real samples.

Figure 11 presents the QQ-Plot of the length of the simulated loss bursts based on the related works compared to the data of the real sample observed in the networks. Observing these comparisons, including results from Table 6, it is possible to identify and confirm that most of the models tested can represent the length of the loss bursts, however, only up to a certain maximum length. From this limit, the simulated models become inefficient to represent the real behavior of the Wifi networks. The hybrid model that uses the GE model and also a heavy-tailed distribution (GE + heavy-tailed) to model the losses is able to present bursts greater than the length of 1500 in which it was also identified in the real sample, however, it still failed to adequately represent the total behavior of the bursts when observing the QQ-Plot. We consider that the tested and simulated models can adequately represent the real samples up to a certain threshold. However, burst sizes greater than this threshold must be modeled using another probability distribution, as we propose in this new packet loss model, which proved to be better in relation to other existing models.

## 5. Conclusions

In this article, we present a new model for packet loss in Wi-Fi networks using the hidden Markov model. The proposed model jointly uses two important wireless network variables: signal-to-noise ratio and channel occupation, which are the ones most correlated with packet loss in Wi-Fi networks. Through clustering techniques, we identified that it is not necessary to use a large number of states for the Markov chain—this is possible due to the simultaneous use of the two observable variables. Unsupervised training was carried out with a data set sampled on a real network, covering a wide variation in network load and the channel’s SNR. The resulting model is more robust than existing models because it is capable of predicting losses in a wide variety of situations without the need for reparametrization. The simulated results, compared with a validation data set, demonstrate that the proposed model is capable of mimicking the characteristics of real networks. Moreover, they show that the proposed model is not only better, but it also significantly outperforms existing models.

## Figures and Tables

**Table 1:** Parameters of related works and proposed model.

Characteristics	[24]	[10]	[11]	[19]	[25]	[26]	[23]	[27]	[8]	Proposed Model
IEEE 802.11	✔	✔	✔	-	✔	✔	✔	-	✔	✔
SNR	✔	-	-	-	✔	-	-	-	-	✔
Channel Occupation (%)	-	-	-	-	-	-	-	-	-	✔
HMM	-	-	✔	✔	-	-	✔	-	-	✔
Heavy-tail distribution	-	-	-	-	✔	-	-	-	✔	✔

**Table 2:** Access Point configuration.

AP Information	Description
AP characteristics	300 Mbps Wireless N ADSL2+ Modem Router
Internet Service Provider	Bridge mode
Channel number	Fixed for each collection (1, 6, or 11)
Transmitter power	Maximum
Wi-Fi standard	IEEE 802.11 b + g + n (auto)
Channel Bandwidth	11b/g – 20 MHz or 11n – 20/40 MHz (auto)

**Table 3:** Packet loss rate in each state.

State	PLR	Standard Deviation	Classification
1	60.97%	24.93%	B
2	0.55%	4.09%	G
3	2.02%	8.36%	I1
4	12.78%	21.76%	I2

**Table 4:** Estimated parameters for BLL.

State	Distribution	Estimated Parameters
B	Pareto type II +Exponencial	α=3.21 e λ=12.32 μ=1682.61
G	Pareto type II +Exponencial	α=3.19 e λ=6.33 μ=44.36
I1	Pareto type II	α=3.42 e λ=7.23
I2	Pareto type II	α=2.07 e λ=4.94

**Table 5:** BLL of validation set and model simulation.

State	Validation Data Set		Model Simulation	
	**Average**	**Std. Dev.**	**Average**	**Std. Dev.**
B	5.67	34.44	5.80	28.64
G	3.00	5.75	2.99	6.01
I1	3.03	4.70	2.97	4.48
I2	4.66	11.04	4.56	11.89

**Table 6:** Comparison of related works with the proposed model.

Model	Average BLL	Maximum BLL	Std. Dev. BLL	MSE
Validation data set	5.37	8853	31.68	0
GE	5.36	65	4.832	0.352
[24]	4.88	54	4.52	0.319
[10]	5.37	158	7.53	0.352
[26]	6.52	96	6.01	0.352
[8]	8.11	11,074	24.08	2.907
[27] 4 states	16.76	204	15.78	0.352
[27] 5 states	22.52	273	21.45	0.352
[27] 7 states	34.85	489	33.80	0.351
[27] 10 states	54.19	721	53.39	0.349
[27] 20 states	150.5	2286	156.67	0.318
Proposed model	5.52	7728	29.75	$0.70\times 10^{-4}$

## Data Availability

Not applicable.
